# Accuracy in Copy Number Calling by qPCR and PRT: A Matter of DNA

**DOI:** 10.1371/journal.pone.0028910

**Published:** 2011-12-13

**Authors:** Nora Fernandez-Jimenez, Ainara Castellanos-Rubio, Leticia Plaza-Izurieta, Galder Gutierrez, Iñaki Irastorza, Luis Castaño, Juan Carlos Vitoria, Jose Ramon Bilbao

**Affiliations:** 1 Immunogenetics Research Laboratory, Cruces University Hospital, Barakaldo, Basque Country, Spain; 2 Department of Genetics, Physical Anthropology and Animal Physiology, University of the Basque Country, Leioa, Basque Country, Spain; 3 Department of Pediatrics, University of the Basque Country, Bilbao, Basque Country, Spain; St. Georges University of London, United Kingdom

## Abstract

The possible implication of copy number variation (CNV) in the genetic susceptibility to human disease needs to be assessed using robust methods that can be applied at a population scale. In this report, we analyze the performance of the two major techniques, quantitative PCR (qPCR) and paralog ratio test (PRT), and investigate the influence of input DNA amount and template integrity on the reliability of both methods. Analysis of three genes (*PRELID1*, *SYNPO* and *DEFB4*) in a large sample set showed that both methods are prone to false copy number assignments if sufficient attention is not paid to DNA concentration and quality. Accurate normalization of samples is essential for reproducible qPCR because it avoids the effect of differential amplification efficiencies between target and control assays, whereas PRT is generally more sensitive to template degradation due to the fact that longer amplicons are usually needed to optimize sensitivity and specificity of paralog sequence PCR. The use of normalized, high quality genomic DNA yields comparable results with both methods.

## Introduction

Copy number variation (CNV) has emerged as a common source of genomic diversity in humans and it is thought to affect at least 12% of the human genome [1]. Rare alterations in gene copy content identified by robust techniques like comparative genomic hybridization (CGH) have been implicated in several developmental diseases and cancer, but more common structural variation of the genome has been proposed to be associated with increased risk to complex diseases, and there is growing interest in population screening of CNVs. It is known that CNVs cannot be efficiently tagged by nearby SNPs because of the possibility of recurrent mutations and transposition of the duplicated genomic segments into new genomic locations [2]. Additionally, genetic association projects that rely on case-control comparisons are particularly vulnerable to inaccuracies in raw data, and may result in false positive evidence of association between CNVs and disease [3]. Thus, direct, reliable methods for CNV assessment that are applicable to large-scale studies are necessary.

PCR-based methods assign gene copy number values according to the ratio of test/reference product yields. In the paralog ratio test (PRT) a single pair of primers is designed to exploit sequence similarities between elements (often dispersed repeats) present both in the copy variable unit (the ‘test’ locus) and at another genomic location that is invariable (the ‘reference’ locus). This strategy avoids the problems caused by the comparison between the yields of two dissimilar amplicons that may have different amplification efficiencies [4]. PRT is indeed a robust, high throughput approach for the study of common CNV at the population level, but identification of a suitable paralog for each target gene is time-consuming (and sometimes impossible) and careful design of primers is necessary before the actual experiment can be performed. In turn, quantitative PCR (qPCR) compares threshold cycles (Ct) between the target gene and an unrelated reference sequence that does not vary in copy content, to generate ΔCt values which are used for CNV calculation. In theory, this is a straightforward strategy that has been used for large-scale CNV analysis to detect disease associations, including the β-defensin cluster and Crohn's disease [5], [6], psoriasis [7] or celiac disease (CD) [8]. However, the ΔCt method is highly dependent on the amplification efficiency of each of the two different assays that are competing in a single reaction. It has been shown that a 4% change in amplification efficiency could result in an error of up to 400% in ΔCt calculation [9] and CNV results obtained by qPCR have been questioned [10].

In this work, we present qPCR as a simple, fast and reliable alternative for CNV analysis if normalized amounts of input template DNA are used. We also investigate the effect of DNA quality in qPCR and PRT-based CNV analysis and compare the performance of both methods. For this purpose we selected 3 genes: *PRELID1*, a gene involved in mitochondrial apoptosis in human primary Th2 cells [11], *SYNPO*, which has been shown to regulate the actin-based shape and motility of dendritic cells [12] and *DEFB4*, a gene that takes part in the innate immune response and is located in the copy number variable β-defensin cluster, previously associated with several autoimmune diseases [13]. Our interest in *PRELID1* and *SYNPO* is due to the fact that they map to putative CNV regions [1] and are potentially implicated in celiac disease pathogenesis because they are located in a CD linkage region and show altered expression in active patient mucosa [14].

## Methods

### Ethics Statement

Human blood samples were collected for immune gene copy number association studies in celiac disease, after written informed consent had been obtained from donors or their parents. This study was approved by the Clinical Trials and Ethics Committee of Hospital de Cruces.

### DNA samples

Genomic DNA was extracted from whole human blood using Nucleospin Blood DNA extraction kit (Macherey-Nagel, Düren, Germany) following the manufacturer's instructions, and resuspended in ddH_2_O. To prepare the normalized sample set, DNA was quantified using Quant-it PicoGreen dsDNA reagent (Invitrogen, Carlsbad, CA) and DNA concentrations were adjusted to 2.5 ng/µl with a Biomek NX^P^ Laboratory Automation Workstation (Beckman Coulter, Fullerton, CA). Non-normalized samples were resuspended in 50 µl ddH_2_O, regardless of DNA concentration. DNA integrity was tested by electrophoresis in 1% agarose-TAE gels.

### Copy number assignment using real time qPCR

Quantitative PCR analysis of *PRELID1* and *SYNPO* gene content was performed in 400 normalized and 400 non-normalized DNA samples using commercially available, predesigned TaqMan Copy Number Assays (Assay IDs: Hs01090614_cn and Hs00669480_cn for *PRELID1* and *SYNPO*, respectively, each consisting of a pair of unlabeled primers and a FAM labeled, MGB probe) and the *RNase P* Copy Number Reference Assay, with a VIC-labeled TAMRA probe (all from Applied Biosystems, Foster City, CA). Experiments were prepared with the Biomek NX^P^ automated liquid handler in 384 microwell plates, and consisted of 10 µl reactions containing 2 µl DNA (from the normalized or non-normalized sample sets), 5 µl Taqman Genotyping Master Mix (Applied Biosystems) and 0.5 µl each of one target gene and reference CNV assay mixes. The *PRELID1* qPCR assay was additionally run in 96 poorly preserved DNA samples, in order to check the impact of DNA quality in copy number assignment. In the case of *DEFB4*, qPCR was carried out in triplicate in 366 normalized genomic DNA samples. Reactions (10 µl) were prepared in the same manner except that a custom primer-probe set was used as the target assay, as previously described [8]. Following the manufacturer's instructions, all qPCR reactions were run in triplicate on an ABI 7900HT instrument (Applied Biosystems) and thermal cycling conditions were 95°C, 10 min followed by 40 cycles of 95°C for 15 s and 60°C for 1 min.

### Copy number assignment using paralog ratio test (PRT)

**Figure 1:**
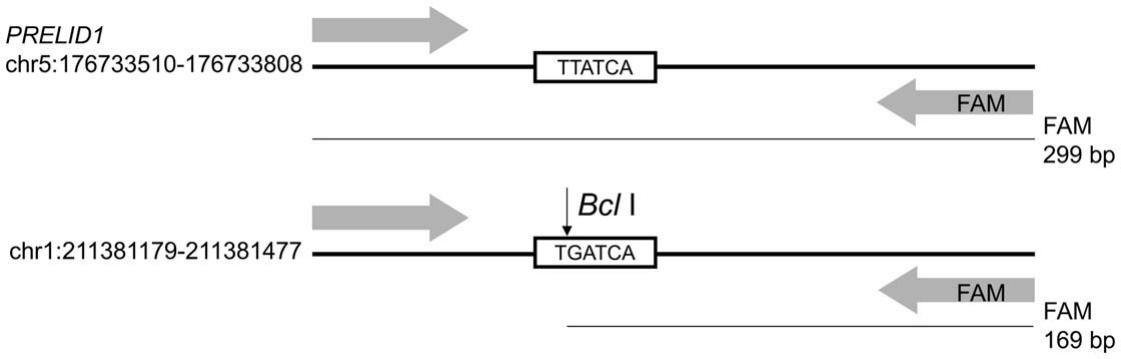
Schematic representation of the PRT assay for *PRELID1*. Forward and FAM-labeled reverse primers amplify two 299 bp fragments from different genomic locations (*PRELID1*, the target gene on chromosome 5 and a paralog on chromosome 1). *Bcl* I digestion results in a 169 bp labeled fragment in the paralog amplicon, which can be distinguished from the undigested *PRELID1* fragment by fluorescent capillary electrophoresis. Coordinates correspond to GRCh37/hg19 genome assembly.

We were not able to design a PRT assay for *SYNPO* because we did not find a suitable invariable copy number paralog for this gene. However, we identified a paralog for *PRELID1* in chromosome 1 (Figure 1). PCR was carried out in 25 µl reactions with 5 ng of input genomic DNA, 1 µM each primer (forward: CCAAGGACCTCGCCAGCAA and reverse: 6-FAM -GGCAAGTCACCGCACCTCTGT), 0.5 mM each dNTP, 1.25 U *Taq* DNA polymerase, 2.5 µl 10× NH_4_-based BioTaq buffer and 1.5 mM supplementary MgCl_2_ (all from BIOLINE, London, UK) in 96 good quality and 96 degraded DNA samples. Amplifications consisted of 26 cycles of 95°C for 30 s, 59°C for 30 s and 72°C for 1 min, to ensure a detectable product yield without reaching amplification *plateau*, followed by a single step of 56°C for 5 min and 72°C for 20 min, to avoid heteroduplex formation. 4 µl of each PCR reaction were added to a digestion mix containing 100 mM NaCl, 50 mM Tris-HCl, 10 mM MgCl2, 1 mM DTT and 15 U *Bcl* I restriction enzyme (New England Biolabs, Ipswich, MA) in order to obtain two FAM-labeled fragments of 299 bp (*PRELID1*) and of 169 bp (paralog in chromosome 1) (Figure 1). After overnight incubation at 50°C, 2 µl of the digestion reaction were mixed with 10 µl HiDi formamide with ROX-500 marker, and analyzed by electrophoresis on an ABI3130XL 36 cm capillary using POP7 polymer (all from Applied Biosystems) and an injection time of 23 s. PRT analyses of *DEFB4* were carried out in 366 normalized samples, as described by Armour et al. [10]. Briefly, PCR was carried out using 5 ng input genomic DNA, 0.5 mM forward primer (CCAGATGAGACCAGTGTCC) and 0.5 mM FAM-labeled reverse primer (TTTTAAGTTCAGCAATTACAGC). Products were amplified using 30 cycles of 95°C for 30 s, 53°C for 30 s and 70°C for 30 s, followed by a single ‘chase’ phase of 53°C for 1 min/70°C for 20 min. Each PCR product was digested with 5 U of *Hae* III (New England Biolabs) and analyzed by electrophoresis, as above.

### Data analysis

The efficiency of the qPCR assays for *PRELID1*, *SYNPO*, *DEFB4* and the endogenous control *RNase P* was calculated using the formula: E = 10^(−1/m)^-1, where *m* is the slope of the function derived from the *Ct versus log-dilution* plot (0.02–200 ng input DNA) of a DNA sample. Analyses of qPCR data were performed using the maximum likelihood method available in Copy Caller v1.0 software (Applied Biosystems), which calculates the probability that the observed data point represents a discrete integer value. These calculations are based solely on ΔCt values, and therefore are highly dependent on target and endogenous control assay efficiencies. Correlation between the starting amount of DNA and Copy Caller-estimated copy number values was calculated using the online tools available at http://danielsoper.com/statcalc3/. In the PRT experiments, a maximum likelihood approach was also used to estimate the copy number values from peak area ratios (target/paralog). In all cases, calculations were performed taking into account that the modal copy numbers of *PRELID1*, *SYNPO* and the β-defensin gene cluster are 2, 2 and 4 [5], respectively. In order to establish the reproducibility of both qPCR and PRT, the analyses of *DEFB4* were repeated twice in the 366 normalized samples and replicate copy number predictions for each sample were compared.

## Results and Discussion

### Copy number assignment by qPCR is affected by input DNA amount

**Figure 2:**
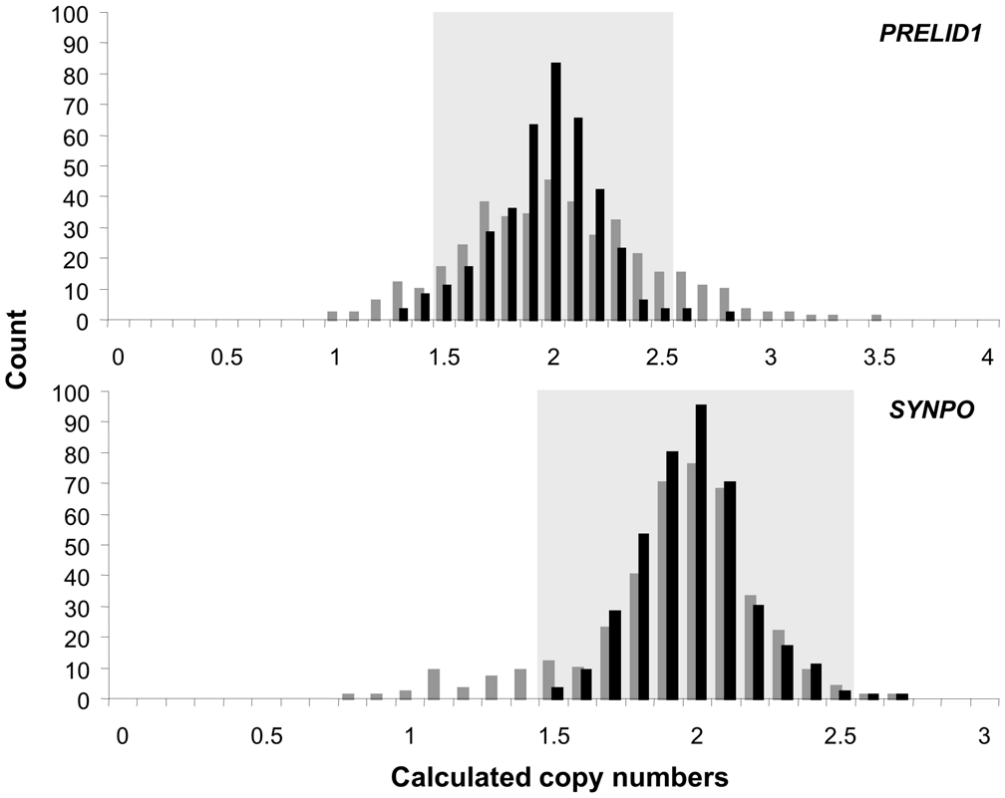
Distribution of *PRELID1* and *SYNPO* calculated copy numbers in normalized (black bars) and randomly diluted (gray bars) DNA samples using qPCR. Samples falling into the light gray areas are predicted to have two copies of the gene.

**Figure 3:**
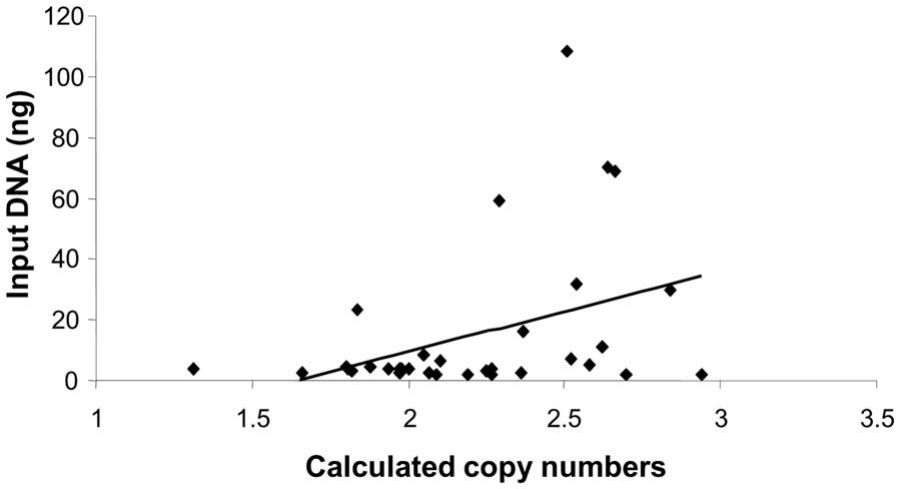
Correlation between the amount of input DNA per qPCR reaction and the raw copy number values for *PRELID1*.

All qPCR amplification plots constructed over a four-log dilution-range of input DNA fitted a straight line (R^2^>0.99). Amplification efficiencies of the *PRELID1* and *SYNPO* assays were 108.23% and 97.84%, respectively, and absolute efficiency differences between each target gene and the internal control (*RNase P*) were 5.95% and 4.42%, respectively (Figure S1). *PRELID1* and *SYNPO* genes were analyzed by qPCR in 400 normalized and 400 non-normalized DNA samples. Calculated copy numbers extracted from Copy Caller software followed a normal distribution that was tightly clustered around 2 copies in the normalized sample cohort. Clustering was less compact in the randomly diluted DNA sample set, where values markedly spread away from the central value, so that gene copy numbers apparently ranged from 1 to 3, for both *PRELID1* and *SYNPO* (Figure 2). In the case of *PRELID1*, there was a significant trend (R = 0.3932; p = 0.0196) towards higher copy number assignments for samples with DNA input amounts above the average (13.28 ng; range 2–120 ng) of the sample set (Figure 3), even after removing outliers (>75 ng input DNA).

Due to the simplicity of its experimental design, qPCR is routinely used for the relative quantitation of mRNA in gene expression analyses, and the same rationale has been transferred to the study of gene copy number variation. However, results obtained with qPCR have not always been robust, and association studies of CNVs with complex human diseases have been conflicting. In fact, the method employed to extract the raw data for copy number determinations relies on calculations based solely on ΔCt values, and assumes that all amplification efficiencies are equal to 100%, or at least equal between the two reactions (target gene and reference sequence) that are simultaneously performed in each experiment [15]. The difference in amplification efficiencies between the two assays used in a qPCR experiment is directly proportional to the difference in the slopes of the serial dilution curves (Figure S1). This difference implies that the distance between the two lines will change as a function of input DNA amount, resulting in proportional changes of ΔCt, and thus of calculated copy numbers.

On the other hand, since PRT uses the same pair of primers for the amplification of both target and paralog sequences, potential differences in amplification efficiency depend only on amplicon structure and sequence, and can generally be obviated if amplicons with quasi-identical sequences are selected (as in the case of *PRELID1*). However, in those cases when length and/or structure of the amplicons differ considerably, attention should be paid to the input DNA amounts for the PCR reactions, in order to avoid inaccuracies in copy number calling.

In a recent study addressing CNV analysis of the β-defensin gene cluster, the qPCR approach was reported to be very sensitive to the quality of template DNA, generating systematic biases that could produce false disease association evidences. However, the authors omitted a crucial issue for robust copy number value assessment because they focused only on template quality, and used a very wide range of starting DNA amounts (10–75 ng per reaction), although differences in efficiency between target and reference genes were close to 5% [16]. Moreover, the authors did not mention which quality factors could be modifying copy number value assignments, and did not provide any evidence to support the importance of DNA quality for copy number analyses by qPCR. On the other hand, in a previous study, it was shown that sample dilution and inhibitor content (such as salts or competing DNA), did not significantly affect amplification efficiency, so that most of its variability relied on primer and amplicon structures and sequences [17]. In our study, we have shown that when the difference in efficiency is between 4 and 6%, variations in input DNA amount can modify ΔCt values and possibly provide false copy number values in *PRELID1* and *SYNPO* (both genes with modal copy numbers of 2). In the case of genes with higher modal copy numbers, this effect would be amplified making calculations more prone to error.

Differences in amplification efficiencies between target and reference amplicons are the biggest challenge to deal with in qPCR optimization for CNV analysis, because they are responsible for variations in ΔCt that can result in artifactual copy number assignments when a wide range of input DNA amounts is used. Our results show that deviation from the average input DNA amount strongly affects final copy number calculations, but that accurate DNA normalization can, at least in part, overcome this problem. Since gene copy numbers are integers, calculated copy number values based on ΔCt results are expected to cluster around discrete figures, and this is best achieved using normalized DNA samples, as observed in this study. If DNA concentrations are normalized, reliable qPCR-based CNV analyses of different genes can be performed using the same reference assay, and primers and probes need to be designed only for each of the target genes of interest or might even be commercially available.

### PRT assays are very sensitive to DNA degradation

**Figure 4:**
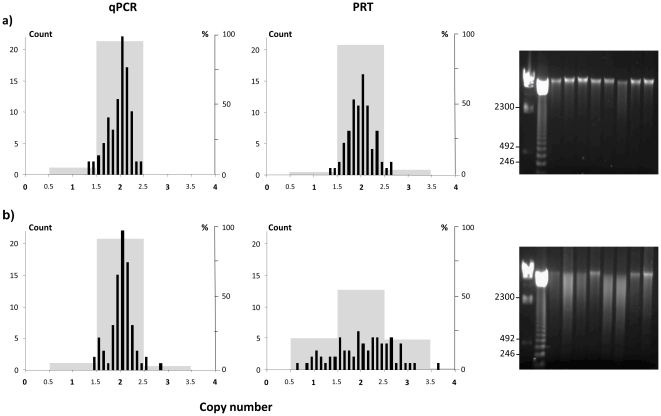
Influence of DNA integrity on *PRELID1* copy number assignment by qPCR and PRT analysis. Distribution of calculated copy number values (black bars) and frequency of predicted copy numbers (gray bars) in a) high quality and b) degraded DNA samples, and gel electrophoresis of a subset of each sample set. Lanes 1 and 2 correspond to DNA size markers (λ-HindIII and 123 bp ladder) and 3–10 to genomic DNA samples. The size of several marker bands is shown in base pairs. The distribution of predicted copy number values differ significantly between good quality and degraded samples in the PRT analysis (χ^2^ = 38.34; p = 2·10^−8^) and between qPCR and PRT copy number assignments in degraded DNA samples (χ^2^ = 33.96; p = 2·10^−7^).

The distribution of calculated *PRELID1* copy numbers using the PRT technique showed a tight Gaussian distribution in good quality DNA samples, but in contrast, degraded DNA samples presented marked spreading over a wider range (<1->3 copy numbers) and did not resemble a normal distribution (Figure 4). PRT has proven to be a robust technique for CNV assignment, but optimal results can be obtained only when high quality DNA samples are used. In turn, DNA degradation does not seem to affect qPCR (Figure 4) and we hypothesize that this lower impact is not caused by any technique-specific condition or characteristic, but could be due to amplicon length, much shorter in the case of the qPCR specific assays compared to PRT assays. *PRELID1* and *DEFB4* amplicon lengths are 107 bp and 127 bp for qPCR and 299 bp and 443 bp for PRT, respectively. The experimental setup of a PRT assay is indeed complicated, because one must design a pair of primers that will amplify two (and not more) genomic stretches of DNA, and this will determine the length of the amplicon. A longer genomic fragment will be repeated less frequently throughout the genome and thus is expected to be more specific, but longer PCR amplifications have been shown to be more sensitive to template DNA degradation [18]. Moreover, as in the case of *SYNPO*, the design of a PRT assay is not always possible, because suitable paralogs are not found for every genomic sequence.

### qPCR and PRT are comparable under controlled conditions

**Figure 5:**
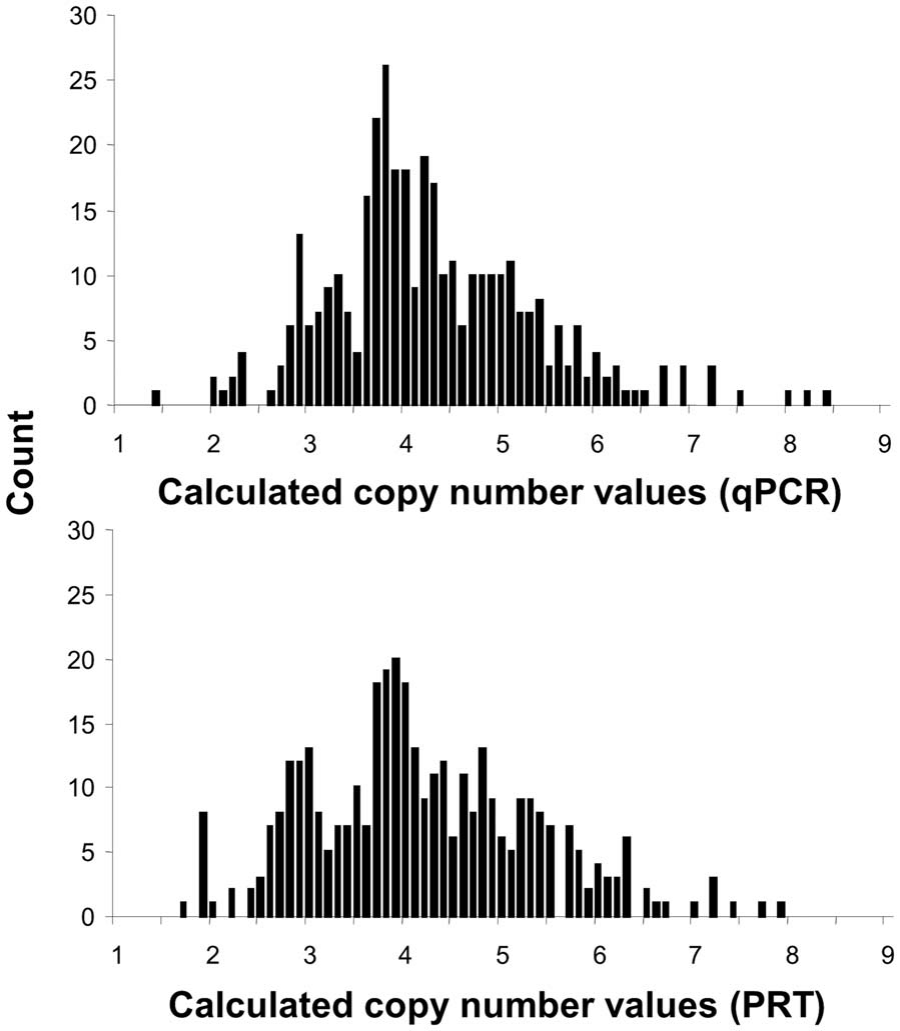
Distribution of calculated copy number values for *DEFB4* obtained with qPCR and PRT in 366 normalized, high quality DNA samples.

**Figure 6:**
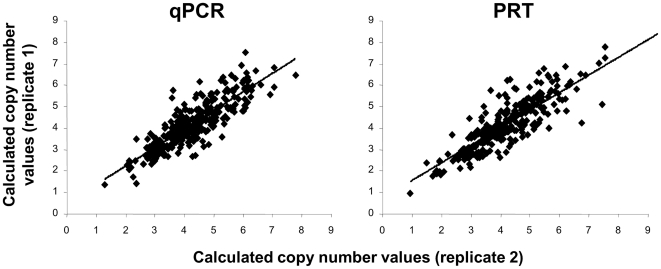
Correlation of calculated *DEFB4* copy number values between replicates in qPCR (R = 0.8546) and PRT (R = 0.8193) techniques.

**Figure 7:**
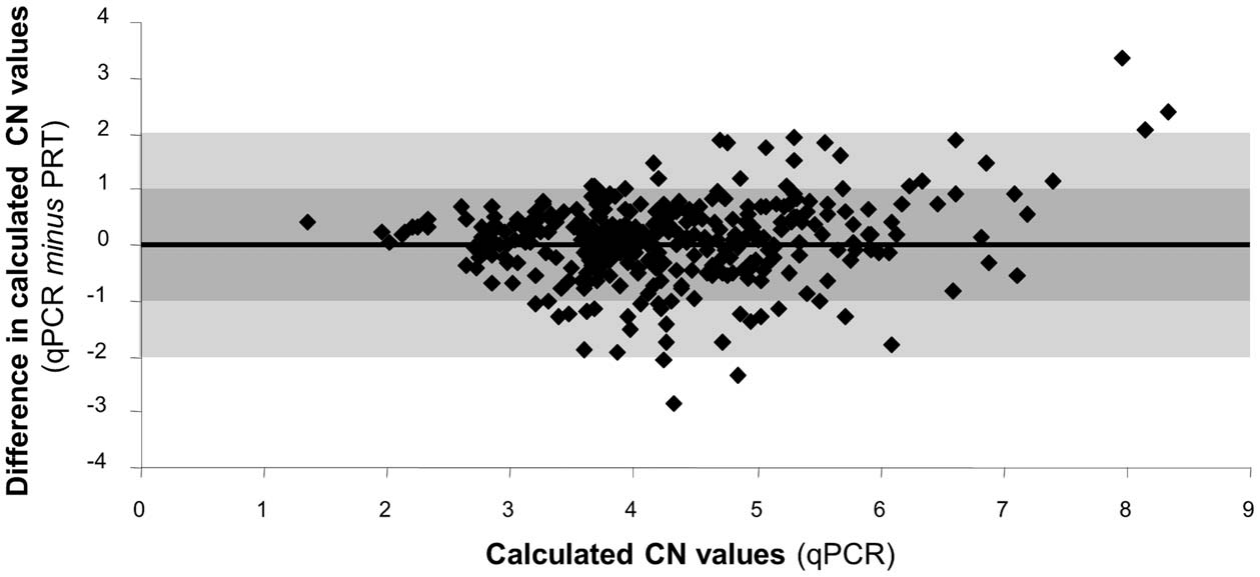
Bland-Altman plot showing deviation of *DEFB4* calculated copy number values obtained by PRT, compared to qPCR.

*DEFB4* gene copy number was analyzed in 366 high quality, normalized DNA samples by both qPCR (absolute efficiency difference with *RNase P* = 7.36%) and PRT and showed a similar distribution of calculated copy numbers, with values clustering around discrete figures (Figure 5). On the other hand, qPCR in triplicate and PRT showed similar standards of reproducibility, and calculated copy numbers showed strong correlation among replicates in both qPCR and PRT (Figure 6). Concordance rates of predicted copy numbers between replicates were 66% and 65%, for qPCR and PRT, respectively. Comparison of the results obtained with the two different techniques also showed a very significant correlation (R = 0.7956) and differences in calculated copy numbers were below 1 in 83% of the samples (Figure 7). Concordance in predicted copy numbers is shown in Table 1; 62% of samples showed the same predicted copy number with both qPCR and PRT, whereas 23% showed a higher copy number prediction with qPCR, and the rest of the sample set (15%) had a lower copy number assignment when analyzed with qPCR.

**Table 1:** Concordance of *DEFB4* gene copy numbers predicted by qPCR and PRT.

copy number		qPCR							
		1	2	3	4	5	6	7	8
**PRT**	**1**	**1**							
	**2**		**9**	5					
	**3**			**50**	33	4			
	**4**			8	**103**	22	2		
	**5**			2	24	**45**	10	2	1
	**6**				4	11	**15**	3	2
	**7**				1	1	2	**4**	
	**8**						1	1	

In brief, our study emphasizes and provides evidence on the extreme importance of DNA normalization when assigning copy number values by qPCR, because this method is sensitive to differences in amplification efficiencies between the target and control assays, and on the relevance of DNA quality when using PRT, due to the fact that longer amplicons are usually needed to optimize sensitivity and specificity, as had already been suggested by other authors [19], especially in large population screenings where the risk for false positive associations is high. Both techniques can be further optimized by analyzing the CNV region more deeply, with the use of multiple primer-probe sets in the case of qPCR [8] or increasing the number of replicates and/or paralog pairs when using PRT [16] to ensure accurate copy number assignment. Under optimal conditions of DNA normalization and quality, both techniques are nearly as comparable between them as they are when compared to their own replicates, and are valid alternatives for population-scale CNV studies.

## Supporting Information

Figure S1 Amplification efficiency plots for Taqman Copy Number assay pairs (target and reference) calculated from multiplex reactions with input DNA concentrations covering 4 orders of magnitude (0.02–200 ng DNA) per reaction.
(TIFF)

## References

[1] Redon R, Ishikawa S, Fitch KR, Feuk L, Perry GH (2006). Global variation in copy number in the human genome.. Nature.

[2] Schrider DR, Hahn MW (2010). Lower linkage disequilibrium at CNVs is due to both recurrent mutation and transposing duplications.. Mol Biol Evol.

[3] Clayton DG, Walker NM, Smyth DJ, Pask R, Cooper JD (2005). Population structure, differential bias and genomic control in a large-scale, case-control association study.. Nat Genet.

[4] Aldhous MC, Abu Bakar S, Prescott NJ, Palla R, Soo K (2010). Measurement methods and accuracy in copy number variation: failure to replicate associations of beta-defensin copy number with Crohn's disease.. Hum Mol Genet.

[5] Fellermann K, Stange DE, Schaeffeler E, Schmalzl H, Wehkamp J (2006). A chromosome 8 gene-cluster polymorphism with low human beta-defensin 2 gene copy number predisposes to Crohn disease of the colon.. Am J Hum Genet.

[6] Bentley RW, Pearson J, Gearry RB, Barclay ML, McKinney C (2010). Association of higher DEFB4 genomic copy number with Crohn's disease.. Am J Gastroenterol.

[7] Hollox EJ, Huffmeier U, Zeeuwen PL, Palla R, Lascorz J (2008). Psoriasis is associated with increased beta-defensin genomic copy number.. Nat Genet.

[8] Fernandez-Jimenez N, Castellanos-Rubio A, Plaza-Izurieta L, Gutierrez G, Castaño L (2010). Analysis of beta-defensin and Toll-like receptor gene copy number variation in celiac disease.. Hum Immunol.

[9] Guescini M, Sisti D, Rocchi MB, Stocchi L, Stocchi V (2008). A new real-time PCR method to overcome significant quantitative inaccuracy due to slight amplification inhibition.. BMC Bioinformatics.

[10] Armour JA, Palla R, Zeeuwen PL, den Heijer M, Schalkwijk J (2007). Accurate, high-throughput typing of copy number variation using paralog ratios from dispersed repeats.. Nucl Acid Res.

[11] Tahvanainen J, Kallonen T, Lähteenmäki H, Heiskanen KM, Westermarck J (2009). PRELI is a mitochondrial regulator of human primary T-helper cell apoptosis, STAT6, and Th2-cell differentiation.. Blood.

[12] Asanuma K, Kim K, Oh J, Giardino L, Chabanis S (2005). Synaptopodin regulates the actin-bundling activity of alpha-actinin in anisoform-specific manner.. J Clin Invest.

[13] Hollox EJ (2008). Copy number variation of beta-defensins and relevance to disease.. Cytogenet Genome Res.

[14] Castellanos-Rubio A, Martin-Pagola A, Santín I, Hualde I, Aransay AM (2008). Combined functional and positional gene information for the identification of susceptibility variants in celiac disease.. Gastroenterology.

[15] Livak KJ, Schmittgen TD (2001). Analysis of relative gene expression data using real-time quantitative PCR and the 2(-Delta Delta C(T)) Method.. Methods.

[16] Fode P, Jespersgaard C, Hardwick RJ, Bogle H, Theisen M (2011). Beta-defensin genomic copy number in different populations: a comparison of three methods.. PLoS One.

[17] Karlen Y, McNair A, Perseguers S, Mazza C, Mermod N (2007). Statistical significance of quantitative PCR.. BMC Bioinformatics.

[18] Putkonen MT, Palo JU, Cano JM, Hedman M, Sajantila A (2010). Factors affecting the STR amplification success in poorly preserved bone simples.. Inves Genet.

[19] Urban TJ, Weintrob AC, Fellay J, Colombo S, Shianna KV (2009). CCL3L1 and HIV/AIDS susceptibility.. Nat Med.

