# Supplementary figures and images for: Accuracy in Copy Number Calling by qPCR and PRT: A Matter of DNA
Source: PLoS One. 2011 Dec 13;6(12):e28910. doi: 10.1371/journal.pone.0028910 (PMC3236783; doi:10.1371/journal.pone.0028910)

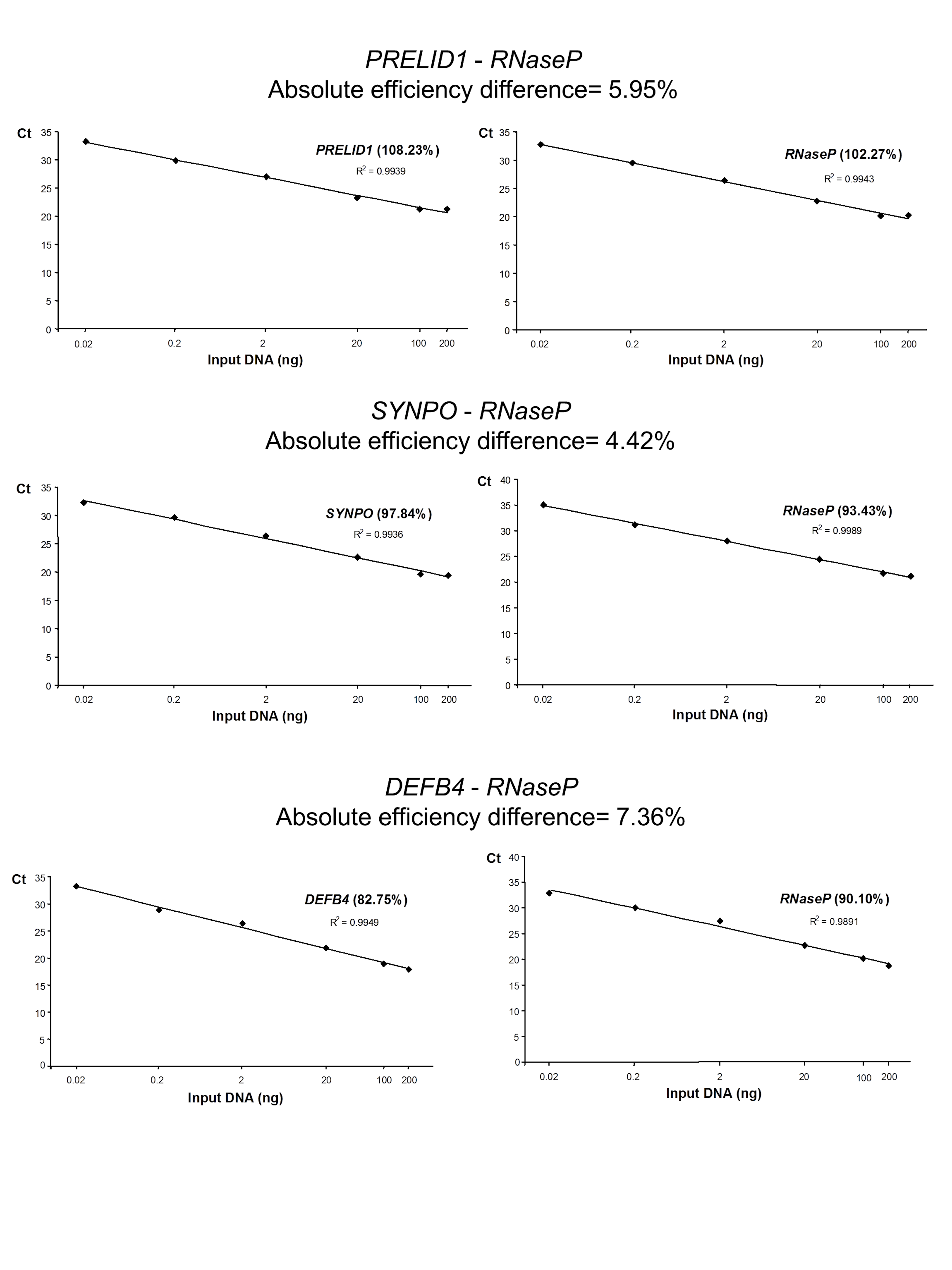

Supplement: Figure S1 — Amplification efficiency plots for Taqman Copy Number assay pairs (target and reference) calculated from multiplex reactions with input DNA concentrations covering 4 orders of magnitude (0.02–200 ng DNA) per reaction. (TIFF) [file pone.0028910.s001.tiff]
